# Effects of Er:YAG laser debonding on changes in the properties of dental zirconia

**DOI:** 10.1371/journal.pone.0313739

**Published:** 2024-11-14

**Authors:** Xinyuan Zhang, Haitao Dong, Xiaomin Wu, Qian Li, Jizhi Zhao, Chunlan Guo

**Affiliations:** Department of Stomatology, Peking Union Medical College Hospital, Chinese Academy of Medical Sciences & Peking Union Medical College, Beijing, China; Kuwait University, Faculty of Dentistry, KUWAIT

## Abstract

**Objectives:**

To investigate changes in the optical and mechanical properties of novel zirconia ceramics applied in dentistry after Er:YAG laser debonding and to evaluate the feasibility and value of reusing zirconia restorations debonded by an Er:YAG laser.

**Methods:**

Four types of zirconia ceramics were investigated: self-glazed zirconia (SGZ), 3Y-TZP, 4Y-PSZ and 5Y-PSZ. Forty rectangular (25 mm*8 mm*1.5 mm) specimens were fabricated for each zirconia type, and a total of 160 specimens were manufactured. The zirconia specimens were divided into four subgroups according to the applied Er:YAG laser debonding process: the control group, 4 W laser group, 5 W laser group, and 6 W laser group. For each subgroup, 10 specimens were subjected to color tests (color difference (△E) and transparency parameter (TP) tests) and subsequent mechanical tests (flexural strength (FS), elastic modulus (EM), Vickers hardness (VH) and surface roughness (Ra) tests). The △E, TP, FS, EM, VH and Ra values were measured and calculated. One random sample from each subgroup was observed by SEM. Statistical analyses were performed by one-way ANOVA followed by post hoc comparisons (α = 0.05).

**Results:**

The △E and TP values after Er:YAG laser debonding were not significantly different among the subgroups (*P* > 0.05). However, the 6 W laser group had the highest △E and lowest TP. The ranges of changes in △E and TP were below the clinically detectable threshold (△E = 1.2, △TP = 1.33). In terms of the mechanical properties, there were no significant differences in the FS, EM, VH or Ra among the subgroups. No obvious microcracks were detected on the surfaces of the zirconia specimens during SEM.

**Conclusions:**

Er:YAG laser debonding does not obviously affect the optical or mechanical properties of novel zirconia ceramics in dentistry. Moreover, it is potentially feasible and valuable to reuse zirconia restorations after Er:YAG laser debonding.

## Introduction

In the last twenty years, monolithic zirconia restorations have been increasingly fabricated for prosthodontic applications [1–4]. Compared with zirconia core ceramics and lithium disilicate glass ceramics, monolithic zirconia ceramics have improved optical properties and excellent mechanical properties [5–7]. However, when the restorations are fractured, mal-positioned, further endodontic therapy are needed, or long-term maintenance of dental implant, the monolithic zirconia restorations are needed to be removable for further treatment. Due to the high strengths of zirconia materials and the high bond strengths of resin cements, removing these materials via the traditional removal method (the use of burs to grind restorations into pieces) is challenging and time-consuming and may cause iatrogenic damage to the tooth tissue, leading to failure of retreatment [8,9].

The erbium-doped yttrium aluminum garnet (Er:YAG) laser debonding method can effectively solve this problem. In the last decade, researchers have increasingly applied Er:YAG lasers to remove glass and zirconia ceramic prosthetics, including ceramic veneers, crowns, and cement-retained implant crowns [8–10]. According to evidence, Er:YAG laser debonding is safe and effective and does not cause iatrogenic damage to tooth tissue; moreover, this approach retains the rebonding area for retreatment [11–13].

Researchers have also investigated the feasibility and value of reusing restorations retrieved by the laser debonding method. Karagoz Yildirak M et al. [14] noted that the rebonding strength of glass ceramic veneers debonded by an Er:YAG laser did not decrease. Deeb JG et al. [15] reported that the retrieval of lithium disilicate crowns from implant abutments using the Er:YAG laser debonding method did not reduce the rebonding efficiency or cause visual damage to the surface. Thus, this approach is of great significance for the treatment of peri-implantitis and the long-term maintenance of cement-retained implant crowns [16]. In a previous study, Er:YAG laser debonding treatment did not affect the mechanical properties of lithium disilicate glass ceramics but did change their optical properties, indicating the possibility of reusing lithium disilicate glass ceramic restorations [17].

To date, with the rapid development of materials in dentistry, novel zirconia ceramics, which are innovative zirconia materials, are being rapidly applied in prosthodontic restorations [1,18]. In some novel zirconia ceramics, the content of Y_2_O_3_ is increased from 3 mol% to 4 mol% or to 5 mol%, hence increasing the cubic phase and transmitting additional light. Examples of these materials include 4 mol% yttria partially stabilized zirconia (4Y-PSZ) and 5 mol% yttria partially stabilized zirconia (5Y-PSZ) [1,18,19]. Self-glazed zirconia (SGZ) is another kind of novel zirconia that is manufactured by three-dimensional (3D) additive manufacturing technology; this material has a small nanograin size and a relatively homogeneous arrangement, leading to increased translucency [20–22]. The novel ceramics not only have better aesthetic properties but also show decreased mechanical strength and toughness [4,18].

However, there is no relevant research on the effects of Er:YAG laser debonding on the properties of zirconia materials. Therefore, the purpose of this study was to evaluate the effects of Er:YAG laser debonding on the optical and mechanical properties of dental zirconia products. The hypothesis was that Er:YAG laser debonding would affect the optical and mechanical properties of zirconia ceramics.

## Materials and methods

### Sample preparation

Four types of dental zirconia ceramics were tested in the study: 3Y-TZP, 4Y-PSZ, 5Y-PSZ and SGZ. Forty rectangular specimens with dimensions of 25 mm*8 mm*1.5 mm in the A1 shade were made for each type of zirconia, and a total of 160 specimens were fabricated. The ceramic types, brand names, main compositions, shades, and manufacturers are presented in Table 1.

**Table 1:** General description about the zirconia materials used in the present study.

Groups	Dental ceramic types	Commercial name	Shade	Main composition (wt%)	Manufacturer
3Y-TZP	3mol% yttria- tetragonal zirconia polycrystal	ST	A1	ZrO_2_: 94.0 Y_2_O_3_: 5.6 Al_2_O_3_: <0.5	Shenzhen Upcera Dental Technology Co
4Y-PSZ	4mol% yttria- partially stabilized zirconia	TT-LT	A1	ZrO_2_: 92.0 Y_2_O_3_: 7.6 Al_2_O_3_: <0.1	Shenzhen Upcera Dental Technology Co
5Y-PSZ	5mol% yttria- partially stabilized zirconia	TT-MT	A1	ZrO_2_: 90.0 Y_2_O_3_: 9.4 Al_2_O_3_: <0.1	Shenzhen Upcera Dental Technology Co
SGZ	Additive 3D gel deposition approach, 3mol% yttria- stabilized tetragonal zirconia polycrystal	Self-Glazed Zirconia	A1	ZrO_2_: 95.0 Y_2_O_3_: 4.5 Others: <0.5	Hangzhou Erran Technology CO. Ltd

3Y-TZP, 4Y-PSZ, and 5Y-PSZ were prepared from presintered blocks by using computer-aided design (CAD)/computer-aided manufacturing (CAM) technology. All 3Y-TZP, 4Y-PSZ, and 5Y-PSZ specimens were milled (Dental Cutting MachineAM-X5, Aidite, Qinhuangdao, China) and sintered in a furnace (Tegra Speed 1500, Teknik Dental, Istanbul, Turkey) according to the manufacturer’s instructions. Afterward, the specimens were glazed (Ivoclar Vivadent, Schaan, Liechtenstein) and exposed to 740 ◦C for 1 min under vacuum in the furnace. The specimens were then ultrasonically cleaned with distilled water and stored dry at room temperature for further evaluation.

SGZ was first constructed by an additive 3D gel deposition approach. The samples contained one hierarchically rough surface opposite the smooth self-glazed surface, both of which spontaneously formed during a net-shaped formation process on the basis of the hybrid gelation principle. All the samples were pressure-less sintered in a muffle furnace at 1,450°C for 90 min. Afterward, the samples were cooled in a furnace to room temperature. Finally, the dimensions of the samples were measured and checked using a digital caliper (500–784; Mitutoyo, Kawasaki, Japan).

In each zirconia group, the specimens were divided into 4 subgroups according to the type of Er:YAG laser debonding operation: the control group (n = 10), the 4 W laser group (n = 10), the 5 W laser group (n = 10), and the 6 W laser group (n = 10). For each group, after the corresponding Er:YAG laser operation, the specimens were first subjected to optical tests (color difference (△E) and transparency parameter (TP) tests) and subsequently to mechanical tests (flexural strength (FS), elastic modulus (EM), Vickers hardness (VH) and surface roughness (Ra) tests). After the flexural strength test, the sample was fractured into two main pieces, one for the Vickers hardness test and the other for the surface roughness test. One random sample from each subgroup was observed by scanning electron microscopy (SEM).

### Laser debonding treatment

An Er:YAG laser (LightWalker, Fotona, Ljubljana, Slovenia) with a sapphire tip 1.3 mm in diameter and 6.5 mm in length was used for laser irradiation. Each laser-treated ceramic specimen was evenly scanned for 300 s, and the laser tip was nearly vertical to the surface at a distance of 5 mm. The laser parameters were as follows. The control group received no Er:YAG laser debonding treatment. The 4 W laser group was set to an energy of 400 mJ, a frequency of 10 Hz, and an energy density of 30.15 J/cm^2^, and the laser was operated in the quantum square pulse (QSP) mode, air spray at 6/8, and water spray at 0/8. The 5 W laser group was set at 500 mJ, 10 Hz, energy density of 37.69 J/cm^2^, QSP mode, air spray at 6/8, and water spray at 0/8. The 6 W laser group was set at 600 mJ, 10 Hz, energy density of 45.23 J/cm^2^, QSP mode, air spray at 6/8, and water spray at 0/8.

### Color test

A Commission Internationale de l’Eclairage (CIE) system and a dental spectrophotometer (Crystaleye, Olympus, Tokyo, Japan) were used in the color tests [17]. The CIE system describes colors based on three parameters: L* represents lightness, a* represents a red–green color, and b* represents a blue–yellow color. A spectrophotometer was used to test each specimen according to the instructions. For each specimen, three different colored backing boards (black, white, and gray) were measured, the L*, a*, and b* values were recorded, and the mean values were calculated.

The color parameters were measured against a gray backing board and were used for the △E tests. The △E values between the control group and the other laser groups (4 W, 5 W and 6 W) were calculated using the following equation, where △L indicates the difference in brightness between the control and laser groups, △a indicates the difference in color changes on the red‒green-axis between the control and laser groups, and △b indicates the difference in color changes on the yellow‒blue-axis between the control and laser groups.


$$△E\;=\;\sqrt{{\left(△L\right)}^{2}+{\left(△a\right)}^{2}+{\left(△b\right)}^{2}}$$

The color parameters were measured against black and white backing boards for the TP tests. The TP value was calculated as follows:

$$TP\;=\;\sqrt{{\left({L_{B}}^{*}-{L_{W}}^{*}\right)}^{2}+{\left(a_{B}-{a_{W}}^{*}\right)}^{2}+{\left({b_{B}}^{*}-{bw}^{*}\right)}^{2}}$$

where the subscript B represents that the color parameters were measured against a black backing board and the subscript W represents the white backing board.

### Flexural strength and elastic modulus tests

Flexural strength tests were performed according to the International Organization for Standardization (ISO) standard 6872 [23]. The standard three-point bending test was carried out using a universal testing machine (INSTRON 5966, INSTRON, Massachusetts, USA). Each sample was placed in the center of two supports with a span of 15 mm. A downward load was applied to the sample at a crosshead speed of 1 mm/min until failure occurred. The FS was calculated as follows:$\sigma \;=\;\frac{3FL}{{2bh}^{2}}$ where σ is the flexural strength (MPa), F is the fracture load (N), L is the length of the support span (mm), b is the width of the sample (mm), and h is the sample thickness (mm).

The static elastic modulus (EM) was calculated from the three-point bending results using the following equation: $Em\;=\;\frac{FL^{3}}{4bh_{d}^{3}}$ where Em is the static elastic modulus and d is the deflection corresponding to load F.

### Vickers hardness test

Vickers hardness tests were performed using a digital microhardness tester with internal software (FALCON 511, INNOVATEST, Maastricht, The Netherlands), according to the ASTM C1327-15 standard [24]. Three Vickers indentations were placed on each specimen with a diamond indenter under a force of 9.8 N for 15 s. The mean VH value of each specimen was calculated.

### Surface roughness test

The average Ra values were measured using a profilometer (M300C; Mahr, Gottingen, Germany) with a driver unit (MarSurf RD 18C, Mahr, Gottingen, Germany) and probe (Mahr PHT6-350, Mahr, Gottingen, Germany). Measurements were performed five times for each specimen at five different sites with a diamond stylus with a 2 μm radius, 90° angle, 1.75 mm cutoff length and 0.2 mm/s measuring speed. The measurements indicated the distance between the peak and valley of the sample surface (accurate to 0.02 μm).

### SEM

To evaluate the surface morphology, one specimen from each subgroup was mounted on a metallic stub, coated with gold, and observed by SEM (CS3400, CamScan, Cambridge, UK) at a magnification of 3000×.

### Statistical analysis

The Shapiro‒Wilk test was applied to determine whether the datasets were normally distributed. Since the △E, TP, FS, ME, VH and Ra values exhibited normal distributions, one-way analysis of variance (ANOVA) was used for analysis and post hoc comparisons were made with the Bonferroni correction (*P* = 0.05). The statistical software program International Business Machines (IBM) Statistical Product and Service Solutions (SPSS) (SPSS Statistics, v22; IBM Corp) was used for data analysis (*P* = 0.05).

## Results

### Optical properties

**Fig 1:**
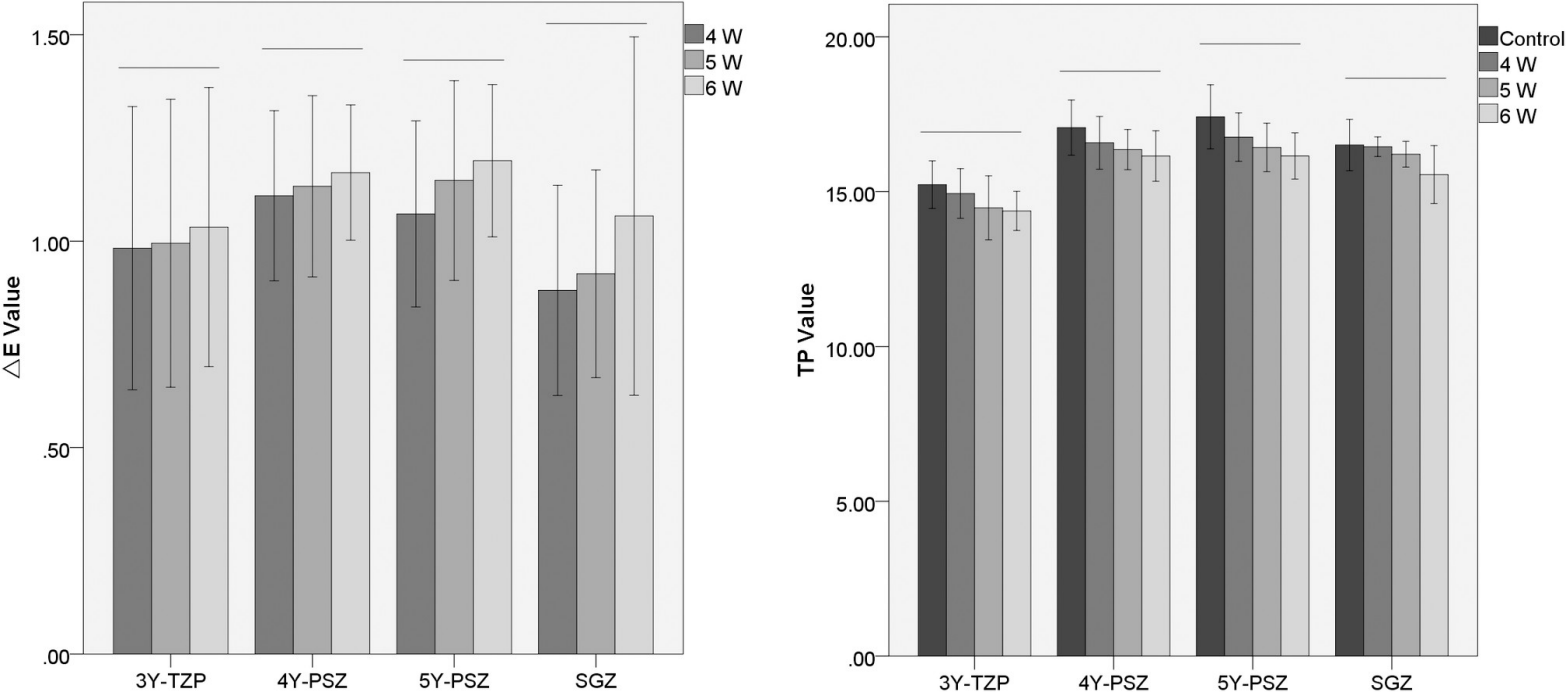
Mean △E and TP values ± SD. The horizontal lines represent no significant differences among subgroups (*P*>0.05).

The mean △E and TP values are presented in Table 2 and Fig 1.

**Table 2:** △E and TP values of the zirconia materials after Er:YAG laser debonding operations (mean ± SD).

		△E	TP
3Y-TZP	Control		15.23(±1.07)
	4 W	0.98(±0.48)	14.94(±1.12)
	5 W	0.99(±0.49)	14.48(±1.44)
	6 W	1.03(±0.47)	14.38(±0.88)
4Y-PSZ	Control		17.07(±1.24)
	4 W	1.11(±0.29)	16.58 (±1.19)
	5 W	1.13(±0.31)	16.36(±0.91)
	6 W	1.17(±0.23)	16.15(±1.42)
5Y-PSZ	Control		17.42(±1.45)
	4 W	1.07(±0.32)	16.76(±1.09)
	5 W	1.15(±0.35)	16.43(±1.09)
	6 W	1.20(±0.26)	16.15(±1.04)
SGZ	Control		16.50(±1.16)
	4 W	0.88(±0.35)	16.45(±0.44)
	5 W	0.92(±0.35)	16.21(±0.58)
	6 W	1.06(±0.61)	15.55(±1.31)

For the △E values, there were no statistically significant differences among the different laser subgroups for the same zirconia material (*P*>0.5). The △E value was positively correlated with the Er:YAG laser energy, and the 6 W subgroup for each zirconia type had the highest △E values. The mean △E values in each subgroup were within the range of the detectable threshold △ E = 1.2 [25].

For the TP values, with the same type of zirconia ceramic, no significant differences were detected among the subgroups (*P*>0.5). However, as the laser energy increased, the TP tended to decrease, and the 6 W subgroup had the lowest TP. The range of changes in transparency was smaller than that of the translucency susceptibility threshold (△ TP = 1.33) [26].

### Mechanical properties

**Fig 2:**
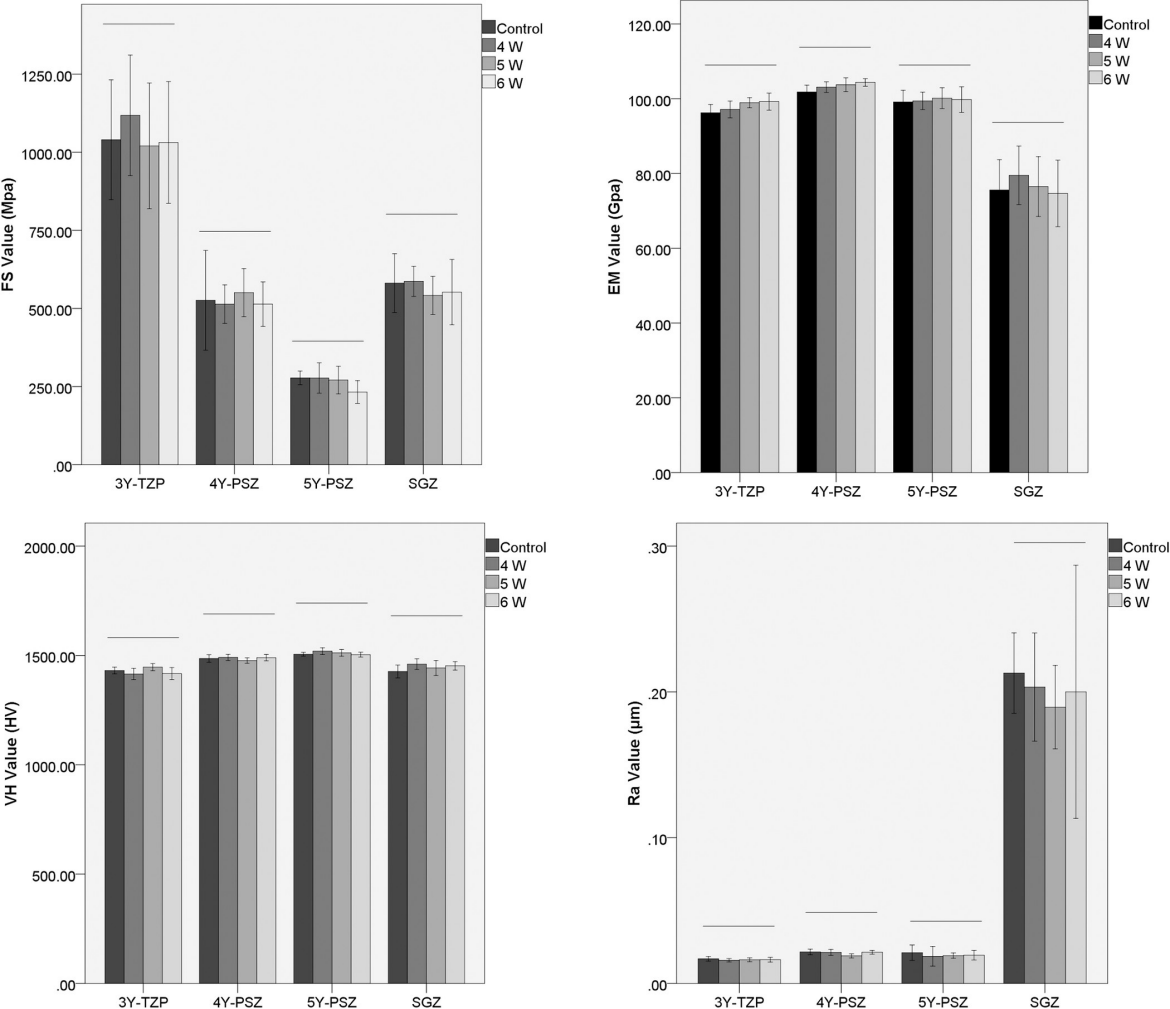
Mean flexural strength (MPa), elastic modulus (GPa), Vickers hardness(HV), surface roughness values ±SD. The horizontal lines represent no significant differences among subgroups (*P*>0.05).

The values of the mechanical properties FS, EM, VH, and Ra are shown in Table 3 and Fig 2.

**Table 3:** Flexural strength, elastic modulus, Vickers hardness and surface roughness values of the zirconia materials after Er:YAG laser debonding operations (mean ± SD).

		FS (MPa)	EM (GPa)	VH (HV)	Ra (μm)
3Y-TZP	Control	1040.14 (±268.02)	96.25 (±3.05)	1430.99 (±221.42)	0.017 (±0.002)
	4 W	1118.32 (±269.74)	97.14 (±3.15)	1414.96 (±35.83)	0.016 (±0.002)
	5 W	1020.36 (±281.39)	98.93 (±1.87)	1446.33 (±223.03)	0.016 (±0.002)
	6 W	1031.37 (±272.32)	99.22 (±3.20)	1417.01 (±238.54)	0.016 (±0.002)
4Y-PSZ	Control	525.97 (±223.25)	101.82 (±2.55)	1486.00 (±24.30)	0.022 (±0.003)
	4 W	513.70 (±86.25)	103.10 (±1.99)	1491.11 (±19.68)	0.021 (±0.003)
	5 W	550.80 (±107.27)	103.73 (±2.57)	1476.72 (±17.57)	0.019 (±0.002)
	6 W	513.83 (±99.53)	104.36 (±1.42)	1489.94 (±20.62)	0.021 (±0.002)
5Y-PSZ	Control	277.72 (±30.51)	99.14 (±4.35)	1505.47 (±12.18)	0.021 (±0.007)
	4 W	277.52 (±68.08)	99.43 (±3.27)	1519.64 (±221.43)	0.019 (±0.009)
	5 W	270.88 (±61.72)	100.14 (±3.89)	1511.84 (±221.57)	0.019 (±0.003)
	6 W	232.55 (±51.12)	99.77 (±4.79)	1503.55 (±215.60)	0.019 (±0.005)
SGZ	Control	581.12 (±131.47)	75.59 (±11.34)	1426.62 (±40.95)	0.213 (±0.039)
	4 W	586.80 (±67.12)	79.51 (±10.95)	1460.20 (±34.43)	0.203 (±0.052)
	5 W	541.75 (±85.40)	76.51 (±11.18)	1442.66 (±47.98)	0.190 (±0.040)
	6 W	552.33 (±146.39)	74.69 (±12.45)	1452.37 (±27.15)	0.200 (±0.121)

The FS, EM, and VH results revealed no significant differences among the control group and the 4–6 W Er:YAG laser debonding subgroups for the same type of zirconia ceramic (*P* > 0.05). For the Ra values, no significant differences among the control group and 4–6 W Er:YAG laser debonding subgroup were detected; however, for the same type of zirconia ceramic specimen (*P* > 0.05), the highest Ra values were reported in the control group (Table 3).

**Fig 3:**
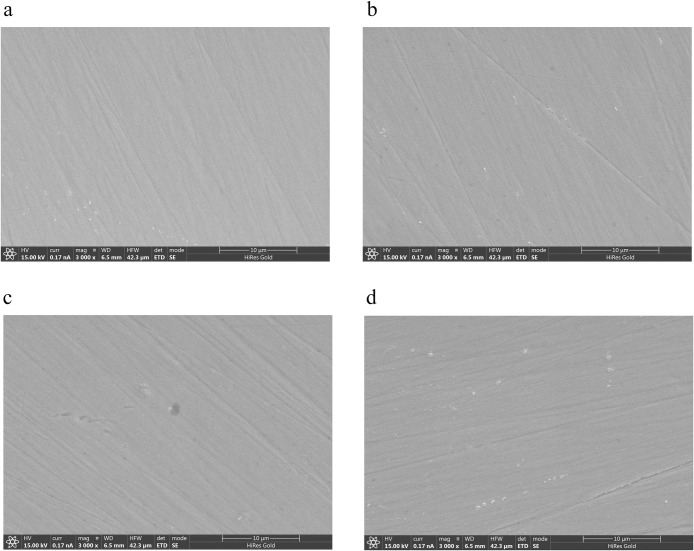
Images of the surface morphology of 5Y-PSZ zirconia specimens under SEM (3000× magnification). a: Control group specimen, b: 4 W laser group specimen, c: 5 W laser group specimen, and d: 6 W laser group specimen.

All the zirconia specimens were intact after laser debonding treatment, and no visible fractures or damage were observed. SEM examinations did not reveal any signs of microcracks or carbonization suggestive of photoablation or thermal ablation. Representative SEM images of the 5Y-PSZ zirconia ceramics at a magnification of 3000× are presented in Fig 3.

## Discussion

Novel zirconia ceramics have increasingly been applied in dental restorations, providing good aesthetic appearance and sufficient mechanical strength [1,4]. However, removing these ceramics was difficult and challenging. Er:YAG laser debonding is considered as an effective, nondestructive and promising method in removing zirconia restorations [8,10]. The removed intact restorations could potentially be reused, which is highly important for the long-term maintenance of restorations, especially implant restorations [11,13]. However, no research has been conducted on the changes in the properties of novel zirconia ceramics after Er:YAG laser debonding. In this study, Er:YAG laser debonding did not significantly affect the optical or mechanical properties of the zirconia ceramics; therefore, the null hypothesis was rejected.

In this study, four kinds of zirconia ceramics were investigated. 3Y-TZP zirconia, which has a flexural strength greater than 1 GPa and a poor aesthetic performance; thus, this material was determined to be unsuitable for anterior monolithic restorations [1,18]. Compared with 3Y-TZP, novel zirconia ceramics can be considered innovative zirconia materials that are increasingly being fabricated for monolithic zirconia restoration; these materials exhibit improved aesthetic performance and adequate strength [2,18]. These novel zirconia products, including 4Y-PSZ, 5Y-PSZ and SGZ, had different phases and arrangements, affecting their nonspecific absorption and the transmittance of light energy, thereby influencing their performance in Er:YAG laser debonding operations [18–20]. On the basis of our previous research, relevant studies on the transmittance of zirconia ceramics with an Er:YAG laser and the underlying factors involved were conducted [27]. In this study, the changes in material properties after Er:YAG laser debonding operations were investigated to evaluate the possibility of reusing different zirconia materials, especially novel zirconia ceramics.

The Er:YAG laser parameters used in this study were based on previous clinical studies and studies simulating clinical Er:YAG laser debonding operations [13,15,17]. The zirconia specimens were not cemented to the tooth structure because mechanical property testing had specific requirements for the dimensions of the ceramic materials in terms of length, width, and height, and no tooth structure satisfied these conditions. Thus, the changes in the properties of the zirconia material itself were investigated, which were not influenced by cementation.

Good aesthetic performance is a prerequisite for the reuse of monolithic zirconia restorations. Both color and transparency are essential for giving prostheses the appearance of natural teeth [7,17,28]. The results of this study revealed that for the same type of zirconia ceramic, Er:YAG laser debonding did not significantly change the TP values. However, it was observed that as the Er:YAG laser energy increased, the TP values tended to decrease. The range of transparency reduction was within the translucency-perceptibility threshold (△ TP = 1.33), indicating that changes could not be detected by observers [26]. Similarly, Turgut et al. [29] reported that 10 W Er:YAG laser surface treatment decreased the translucency of glass ceramic veneers. Kurtulmus-Yilmaz S et al. [30] reported that 4 W Er,Cr:YSGG laser surface treatment slightly decreased the TP of 3Y-TZP zirconia specimens. This decrease occurred because the ceramic surface defect quantity and size and porosity affected the reflection, diffuse reflection and transmission of light energy [31,32]. The ablation and thermal effects of the Er:YAG laser could melt the ceramic surface, altering the texture and roughness and thus changing the ceramic translucency [17,31,32]. Wang et al. analyzed the relationships among surface textures and optical parameters and noted that the smoother the ceramic surface was, the lower the transmittance was [33].

The results of roughness tests also confirmed this viewpoint. On the basis of the roughness results, different laser energies did not cause significant changes in roughness, but there was a certain decreasing trend, which was consistent with the decrease in transparency.

According to the findings, the △E values did not significantly differ among the different laser groups; the value slightly increased with increasing Er:YAG laser energy, and the 6 W laser subgroup in each group presented the highest value. However, all the values were below the CIE L*a*b* perceptibility threshold (ΔE = 1.2) [25], suggesting that laser debonding could change the colors of zirconia ceramics, but these alterations could not be detected by observers. In agreement with the results, Kurtulmus-Yilmaz S et al. reported that 4-W Er,Cr:YSGG laser surface treatment changed the colors of zirconia ceramics at ΔE = 1.4, which was above the CIEDE2000 perceptibility threshold (ΔE = 0.8) but below the unacceptability threshold (ΔE = 1.8) [30]. Moreover, the changes in color could be attributed to the alterations in the surface texture caused by erbium laser irradiation. The differences in the ΔE alteration ranges could be explained by the differences in thickness, ceramic types and experimental and analysis methods [31–33].

With respect to the mechanical properties of dental ceramics, the flexural strength, elastic modulus, Vickers hardness and surface roughness are considered crucial factors for achieving long-term stability and mastication [34–36]. The flexural strength can be described as the ability of dental ceramics to tolerate maximum forces, and it is a key factor for the clinical success of ceramic restorations [30,31,34]. Laser energy can cause heat damage on the zirconia surface, changing the volume and morphology and transforming the phases between the tetragonal and monoclinic phases, leading to alterations in strength and hardness [18,35]. Kurtulmus-Yilmaz S et al. conducted 2–6 W Er,Cr:YSGG laser irradiation in the postsintering stage with a constant cooling procedure (55% water and 65% air) and revealed that 5 W and 6 W laser treatment had the lowest strengths among all laser test groups, while 4 W laser treatment had the highest strength values [35]. The laser groups were significantly different from the control group. In their study, field emission (FE)–SEM results revealed corresponding irregularities on the surfaces of specimens from the 5 W and 6 W laser groups, and X-ray diffraction (XRD) analysis confirmed the transformation between the monoclinic and tetragonal phases. In this study, 4–6 W Er:YAG laser irradiation was conducted on a zirconia surface with 0/8 water and 8/8 air to simulate clinical laser debonding and ensure sufficient efficiency. The results showed that in the SGZ, 5Y-PSZ and 3Y-TZP groups, the 4 W laser subgroup had the highest strength values, while the 5 W and 6 W laser subgroups exhibited lower values than the control group, but the differences among all the groups were not significant. These results were in agreement with the SEM results.

Hardness is a special mechanical property that represents a material’s resistance to penetration, which affects material degradation, leading to fatigue and decreasing the survival rate of materials [17,24,34]. Several researchers have indicated an obvious correlation between a decrease in hardness and an increase in the monoclinic phase of zirconia [35]. In this study, the Er:YAG laser debonding operation had no influence on the VH of zirconia ceramics, which was in good agreement with the findings of other scholars [34]. There is a lack of studies regarding the changes in the hardness of zirconia ceramics after laser irradiation, and this study contributes to the literature.

Surface roughness is a factor involved in bacterial adhesion and may affect the wear of the opposing dentition and optical properties [36,37], thus influencing the long-term stability of restoration use. Goknil Ergun Kunt [38] applied Er:YAG laser irradiation to zirconia ceramics and reported a slight increase in the mean surface roughness, but these differences were not significant. Moreover, in their study, these scholars detected small fissures and narrow microcracks on the surfaces through SEM. Compared to this research, the control group had the highest Ra, while no obvious differences were observed among the subgroups. Similar morphologies were observed on the specimen surfaces in the SEM images, which were consistent with the mechanical and optical results. This could be because the heat increase during laser irradiation may cause melting of the materials’ surfaces, resulting in decreased roughness and translucency [17,32].

In this in vitro study, experimental evidence for the reuse of Er:YAG laser-removed zirconia restorations were provided. This study had several limitations. As mentioned above, due to the size requirements of the specimens used in optical and mechanical experiments, the specimens could not be bonded to dental tissue to simulate clinical laser debonding operations; thus, changes in the properties of the zirconia material itself were investigated, which was not affected by cementation. Another limitation is that we did not evaluate the rebonding strength of zirconia ceramics after Er:YAG laser debonding. However, further studies are needed to observe the clinical efficacy and performance of removing zirconia restorations via the Er:YAG laser.

## Conclusions

Within the limitations of this study, the following conclusions could be drawn: Er:YAG laser debonding did not change the optical or mechanical properties of dental zirconia ceramics, even those of novel zirconia ceramics. Moreover, this process did not cause any microcracks to form on the surfaces of the zirconia ceramics. The results provide important evidence for the reuse of zirconia restorations in clinical practice and for the clinical application of Er:YAG laser debonding operations.

## Supporting information

S1 Raw data (XLSX)
